# The adhesion and migration of microglia to β-amyloid (Aβ) is decreased with aging and inhibited by Nogo/NgR pathway

**DOI:** 10.1186/s12974-018-1250-1

**Published:** 2018-07-20

**Authors:** Yinquan Fang, Jianing Wang, Lemeng Yao, Chenhui Li, Jing Wang, Yuan Liu, Xia Tao, Hao Sun, Hong Liao

**Affiliations:** 1 Jiangsu Key laboratory of Drug Screening, China Pharmaceutical University, 24 Tongjiaxiang Street, Nanjing, 210009 China; 2 Department of Pharmacology, Jiangsu Key Laboratory of Neurodegeneration, Nanjing Medical University, Nanjing, Jiangsu China

**Keywords:** Nogo, Nogo receptor, Microglia, Aβ, Adhesion, Migration, Alzheimer’s disease

## Abstract

**Background:**

Alzheimer’s disease is characterized by progressive accumulation of β-amyloid (Aβ)-containing amyloid plaques, and microglia play a critical role in internalization and degradation of Aβ. Our previous research confirmed that Nogo-66 binding to Nogo receptors (NgR) expressed on microglia inhibits cell adhesion and migration in vitro.

**Methods:**

The adhesion and migration of microglia isolated from WT and APP/PS1 mice from different ages were measured by adhesion assays and transwells. After NEP1-40 (a competitive antagonist of Nogo/NgR pathway) was intracerebroventricularly administered via mini-osmotic pumps for 2 months in APP/PS1 transgenic mice, microglial recruitment toward Aβ deposits and CD36 expression were determined.

**Results:**

In this paper, we found that aging led to a reduction of microglia adhesion and migration to fAβ_1–42_ in WT and APP/PS1 mice. The adhesion and migration of microglia to fAβ_1–42_ were downregulated by the Nogo, which was mediated by NgR, and the increased inhibitory effects of the Nogo could be observed in aged mice. Moreover, Rho GTPases contributed to the effects of the Nogo on adhesion and migration of microglia to fAβ_1–42_ by regulating cytoskeleton arrangement. Furthermore, blocking the Nogo/NgR pathway enhanced recruitment of microglia toward Aβ deposits and expression of CD36 in APP/PS1 mice.

**Conclusion:**

Taken together, Nogo/NgR pathway could take part in Aβ pathology in AD by modulating microglial adhesion and migration to Aβ and the Nogo/NgR pathway might be an important target for treating AD.

**Electronic supplementary material:**

The online version of this article (10.1186/s12974-018-1250-1) contains supplementary material, which is available to authorized users.

## Background

Alzheimer’s disease (AD) is the most frequent neurodegenerative disorder and the leading cause of dementia worldwide [1], progressing from minor memory problems to complete loss of cognitive functions and eventually death. Recently, many studies have found that neuroinflammation is one of the causes of AD [2]. Microglia, the primary immune cells of the brain, play a central role in the pathogenesis of AD.

Microglia have a biphasic neurotoxic-neuroprotective role in the pathogenesis of AD [3]. For instance, over-activated microglia can release proinflammatory cytokines and neurotoxic molecules, which can exacerbate disease [4–6]. Conversely, microglia also migrate to, adhere to, and phagocytose Aβ, which could help clear Aβ plaques from the brain parenchyma [7–9]. Interestingly, microglia revealed an age-dependent decrease in ability to phagocytose Aβ fibrils and expression of Aβ-interacting protein is decreased in vitro [10]. Moreover, in line with progression of AD pathology, proinflammatory cytokines reduce expressions of Aβ-binding receptors and Aβ-degrading enzymes and further decrease ability of microglial Aβ clearance [11].

As a member of the reticulon family, Nogo-A inhibits axonal extension and fibroblast spreading in CNS disease [12, 13]. The Nogo receptor (NgR) is a neuronal surface glycosyl phosphatidylinositol (GPI)-linked receptor, which binds to Nogo-66 (a hydrophilic 66 amino acids long region of Nogo-A) with high affinity [14, 15]. Some studies reveal that Nogo and NgR are involved in the pathogenesis of AD. For example, expressions of Nogo-A and NgR are increased in patients with AD and in aged rats with deficits of spatial cognition [16–18]. Furthermore, Nogo and NgR are associated with Aβ [16, 19], and it has been demonstrated that deleting Nogo ameliorates learning and memory deficits in APP transgenic mice [20], and subcutaneous NgR(310)ecto-Fc treatment reduces brain Aβ plaque load and improves spatial memory in APPswe/PSEN-1ΔE9 transgenic mice [21].

Our previous research has confirmed that NgR is expressed on microglia and that Nogo-66 bindings with NgR could inhibit adhesion and migration of microglia through the RhoA/Rho associate kinase (ROCK) pathway in vitro [22]. The interaction of Nogo-P4 with NgR elevates the expressions of proinflammatory enzymes (iNOS and COX-2) and cytokines (IL-1β, TNF-α, NO, and PGE2) in primary microglia [23], and neuroinflammation mediated by the Nogo/NgR pathway in microglia plays a role in the formation of Aβ plaques in vitro and in vivo [24]. Moreover, with aging, the increased expression of NgR in microglia has been reported [25]. These findings provide a new insight into whether the Nogo participate in the adhesion and migration of microglia to Aβ. In this study, we found that as aging microglia exhibited decrease in adhesion and migration to fAβ_1–42_ in vitro. The interaction of Nogo with NgR inhibited the adhesion and migration of microglia to Aβ, in which adhesion and migration are important processes of phagocytosis. Furthermore, cytoskeleton reorganization mediated by Rho GTPases also contributed to the effects of the Nogo on the adhesion and migration of microglia to Aβ. Moreover, the blocking of the Nogo/NgR pathway enhanced microglial recruitment toward Aβ deposits and expression of CD36 in APP/PS1 mice.

## Methods

### Animals

C57BL/6J mice were obtained from Vitalriver (Beijing, China). APP/PS1 transgenic mice were purchased from the animal model center of Nanjing University (Nanjing, China), and the mice were generated from the B6C3-Tg (APPswe, PSEN1dE9) 85Dbo/J double transgenic mouse line (stock no. 004462) provided by the National Jackson Animal Center (Bar Harbor, Maine, USA). All of the mice were raised in a thermostatic 12-h/12-h dark-light cycle environment, with free access to food and water. All animal tests were carried out in accordance with the US National Institute of Health (NIH) Guide for the Care and Use of Laboratory. All experimental procedures were approved by the Institutional Animal Care and Use Committee (IACUC) of the Nanjing Medical University Experimental Animal Department.

### Microglia from neonatal mice

Primary microglia culture were prepared from cerebral cortex of neonatal C57BL/6J mice as described previously [23]. Briefly, meninges were removed from the brains and the cortex was enzymatically dissociated (0.25% trypsin-EDTA, Sigma, St Louis, MO, USA). Then, cells were suspended in Dulbecco’s Modified Eagle’s Medium/nutrient mixture F-12 (DMEM/F12, Gibco, Carlsbad, CA, USA) supplement with 10% FBS (Gibco) and seeded into poly-L-lysine (PLL, 0.01 mg/ml, Sigma) pre-coated T75 tissue culture flasks. After 3, 7, and 10 days, the culture medium was renewed. After 2 weeks in culture, the cells were isolated by gently shaking of the flask. About 95% of these cells were positive for CD11b, a marker for microglia cell types.

### Microglia from adult mice

Three-month-old and 15-month-old C57BL/6J mice and 3-month-old and 15-month-old APP/PS1 mice were anesthetized with chloral hydrate (100 mg/kg, i.p.), and perfused with ice-cold D-Hanks’ balanced salt solution to wash away all contaminating blood cells from the brain. The cerebellum and meninges were removed from the brains. Then, the tissue was dissected and dissociated in an enzymatic solution at 37 °C and 5% CO_2_ and continuously stirred for 90 min. The enzymatic solution [26] contained 116 mM NaCl, 5.4 mM KCl, 26 mM NaHCO_3_, 1 mM NaH2PO_4_, 1.5 mM CaCl2, 1 mM MgSO_4_, 0.5 mM EDTA, 25 mM glucose, 1 mM cysteine, and 20 units/ml papain (Sigma). Next, the enzymatic reaction was quenched by the addition of 20 ml of 20% FBS in D-Hank’s. After centrifugation at 200 g for 7 min at room temperature, the tissues were dissociated in 0.05 mg/ml DNase I (Sigma) in D-Hank’s and incubated for 5 min at room temperature. Then, the tissues were gently disrupted and filtered through a 70-μm cell strainer (Corning, New York, USA). To remove myelin [27], the cells were suspended in 20 ml 20% stock isotonic Percoll (SIP = *v*/*v* ratio: 9/10 Percoll (GE Healthcare, Princeton, NJ, USA) + 1/10 D-Hank’s 10×) in D-Hank’s and centrifuged for 20 min at 500 g with slow acceleration and no brake. The supernatant containing the myelin was removed, and the pelleted cells were washed with D-Hank’s. Then, the cells were incubated with anti-mouse CD11b-coated microbeads (Miltenyi Biotec, Bergisch Gladbach, Germany) for 15 min at 4 °C. The cells were washed with washing buffer (0.5% BSA, 2 mM EDTA in PBS) to remove unbound beads. The cells were resuspended in washing buffer and passed over a magnetic MACS Cell Separation column (Miltenyi Biotec), and the column was rinsed twice with washing buffer. CD11b-positive microglia were eluted by removing the column from the magnetic holder and the pushing washing buffer through the column with a plunger. The cells were centrifuged and washed with DMEM/F12 containing 10% FBS. Approximately 95% of these cells were positive for CD11b, a marker for microglia cell types.

### Western blot analysis

After 2 months of administration, mice were deeply anesthetized with chloral hydrate (100 mg/kg, i.p.). After perfusion with PBS, the brain was quickly dissected and stored at − 80 °C until further use. Snap-frozen brain tissue was homogenized in RIPA buffer (Beyotime Biotechnology Co., Shanghai, China) supplemented with a protease inhibitor cocktail (Roche Molecular Biochemicals, Indianapolis, IN, USA). Extracts were centrifuged at 12,000 g for 20 min at 4 °C, and the supernatant was collected and the protein concentration was determined using bicinchoninic acid protein assay kit (Beyotime Biotechnology).

After different treatments, the cells were lysed in lysis buffer (Beyotime Biotechnology, Nanjing, China) containing a protease inhibitor cocktail (Roche Molecular Biochemicals). Debris were removed by centrifugation, and the supernatant was collected. The protein concentration was determined using bicinchoninic acid protein assay kit (Beyotime Biotechnology).

Equal amounts of protein (30 μg) were separated electrophoretically using denaturing gels and transferred to nitrocellulose membranes. Subsequently, the membranes were blocked for 1 h at room temperature with 5% BSA in TBST and incubated with following primary antibody overnight at 4 °C: rabbit anti-p-Myosin Light Chain 2 (MLC) at Ser19 polyclonal antibody (1:1000; 3671; Cell Signaling Technology Inc., Danvers, MA, USA), rabbit anti-MLC polyclonal antibody (1:1000; 3672; Cell Signaling Technology Inc.), rabbit anti-p-cofilin at Ser3 polyclonal antibody (1:1000; 3311; Cell Signaling Technology Inc.), rabbit anti-cofilin polyclonal antibody (1:1000; 3318; Cell Signaling Technology Inc.), mouse anti-β-actin monoclonal antibody (1:2000; sc-47778; Santa Cruz Biotechnology, MA, USA), rabbit anti-CD36 polyclonal antibody (1:1000; NB400-144; Novus Biologicals, Littleton, CO, USA), rabbit anti-Ras GTPase-activating-like protein (IQGAP) polyclonal antibody (1:1000; ab133490; Abcam, Cambridge, MA, USA). After washed with TBST thrice, the membranes were incubated with horseradish peroxidase-conjugated secondary antibodies anti-mouse IgG (1:10000; RABHRP2; Sigma) or anti-rabbit IgG (1:5000; 7074; Cell Signaling Technology Inc.). The immunoreactive bands were visualized using chemiluminescence reagents (ECL; Millipore, Billerica, MA, USA) and captured with Bio-Rad Gel Doc XR documentation system. Pixel density of bands was performed using Quantity One software.

### Preparation of fibrillar Aβ (fAβ)

Aβ_1–42_ or Aβ_42–1_ (all from AnaSpec Inc., San Jose, California, USA) lyophilized powder was dissolved to 1 mg/200 μl in 100% hexafluoroisopropanol and aliquoted into 10 μl portions, dried in a vacuum centrifuge, and stored at − 20 °C. To fibrillize, Aβ_1–42_ and Aβ_42–1_ peptides were resuspended in sterile ddH_2_O followed by incubation for 1 week at 37 °C [28, 29]. The Congo Red dye binding assay [30] was used to valid the fAβ.

### Adhesion assay

Adhesion assay was performed according to previous described method [22]. The methanol-solubilized nitrocellulose was added into 24-wells for 1s, washed using ddH_2_O, and air-dried under a sterile hood. Two microliter spots of BSA (0.01% in PBS), Rtn-P4 (100 μg/ml), Nogo-P4 (100 μg/ml), fAβ_1–42_ (10 μM), Rtn-P4 (100 μg/ml) + fAβ_1–42_ (10 μM), Nogo-P4 (100 μg/ml) + fAβ_1–42_ (10 μM), or fAβ_42–1_ (10 μM) were applied in duplicate to the nitrocellulose-coated surfaces of the wells and incubated overnight at 4 °C. In the absence or presence of phosphatidylinositol-specific phospholipase C (PI-PLC, 0.3 U/ml, Sigma), NEP1-40 (10 μM, Millipore) or Y27632 [(1)-(R)-trans-4-(1-aminoethyl)-N-(4-pyridyl) cyclo-hexanecarboxamide dihydrochloride] (50 μM, Calbiochem-Novabiochem., San Diego, CA, USA) for 30 min, freshly isolated microglia were seeded in the pre-coated wells for adhesion assay. After 24 h, the plate was washed thrice with PBS, and the cells were fixed with 4% paraformaldehyde and stained with Crystal Violet solution (0.2%). The number of cells adhering to fields which pre-coated by protein spots referred above was randomly counted under microscopy.

### Migration assay

Costar Transwells (polycarbonate filter, 8 lm-pore size, Millipore) were used in the migration assay to examine the ability of microglial migration [22]. The undersurfaces of transwell membranes were coated with BSA (0.01% in PBS), Rtn-P4 (100 μg/ml), Nogo-P4 (100 μg/ml), fAβ_1–42_ (10 μM), Rtn-P4 (100 μg/ml) + fAβ_1–42_ (10 μM), Nogo-P4 (100 μg/ml) + fAβ_1–42_ (10 μM), or fAβ_42–1_ (10 μM) overnight at 4 °C. Freshly isolated microglia untreated or treated with PI-PLC (0.3 U/ml), NEP1-40 (10 μM), or Y27632 (50 μM) for 30 min were suspended in serum-free culture medium and planted into the upper chamber for migration assay. After 24 h, the inserts were fixed with 4% paraformaldehyde and stained with Crystal Violet solution (0.2%). After washing with PBS, cells from the inner surface of the insert were gently wiped out with cotton-tipped swabs, and the cells from the outer surface of the insert were counted under microscope at five fields per filter.

### RhoA, Rac1, and Cdc42 activity assay

To measure the activity of RhoA, Rac1, and Cdc42, 96-well plates were coated with methanol-solubilized nitrocellulose and air-dried under a sterile hood. PLL coating on the nitrocellulose-coated surfaces of the wells was performed to promote microglia adhesion. Then, BSA (0.01% in PBS), fAβ_1–42_ (10 μM), Nogo-P4 (100 μg/ml), or Nogo-P4 (100 μg/ml) + fAβ_1–42_ (10 μM) were coated on the wells overnight at 4 °C. Adult microglia were added into the pre-coated wells and cultured for 6 h.

The activity of RhoA, Rac1, and Cdc42 were determined by commercial Rho Activation Assay Kit (Millipore) and Rac1/Cdc42 Activation Assay Kit (Millipore) following the manufacturer’s instructions.

### F-actin staining

For observing F-actin, microglia were cultured in 96-well plates pre-coated with BSA (0.01% in PBS), fAβ_1–42_ (10 μM), Nogo-P4 (100 μg/ml), or Nogo-P4 (100 μg/ml) + fAβ_1–42_ (10 μM) in the absence or presence of PI-PLC (0.3 U/ml), NEP1-40 (10 μM), or Y27632 (50 μM) for 8 h. Then, the cells were fixed with 4% paraformaldehyde and permeabilized with 0.1% Triton X-100 in PBS. After incubating with 0.5 μg/ml TRITC-phalloidin (Sigma) for 30 min at room temperature, fluorescence was then examined by fluorescence microscopy. For quantification of cell spread, the spread areas were calculated and analyzed using the Image-Pro Plus software. The average spread areas of the cells in each group were expressed as the percentage of spread areas of the BSA group.

### NEP1-40 treatment

To deliver NEP1-40 peptide, 6-month-old male APP/PS1 mice were anesthetized with chloral hydrate (100 mg/kg, i.p.), and a burr hole was drilled on the skull. A cannula (Alzet brain infusion kit II; Alza, Palo Alto, CA) was stereotactically introduced into the right lateral ventricle at the coordinates: 0.6 mm posterior and 1.2 mm lateral to bregma and 2.0 mm deep to the pial surface. The cannula was held in place with cyanoacrylate, and the catheter was attached to osmotic micropump (Alzet 2004, 0.25 μl/h for 28 d; Alza). The pump was placed subcutaneously in the mid scapular area of the back of the mice. Animals were categorized into vehicle (97.5% PBS + 2.5% DMSO, 10 male APP/PS1 mice, 5 mice for Western blot, and 5 mice for immunofluorescence)-treated and NEP1-40 (500 μM in vehicle, 8 male APP/PS1 mice, 4 mice for Western blot, and 4 mice for immunofluorescence)-treated groups. Pumps were replaced after 28 days and connected to the same cannula.

### Tissue preparation

After infusion for 2 months, mice were deeply anesthetized using chloral hydrate (100 mg/kg, i.p.) and perfused intracardially with cold 4% paraformaldehyde (PFA) in PBS followed by perfusion with PBS. Brain tissues were fixed with PFA overnight at 4 °C and equilibrated by immersing in 15 and 30% sucrose in PBS overnight at 4 °C, respectively. Brain tissues were then sectioned to a thickness of 15 μm on Leica-1900 cryostat (Leica Instruments, Germany). Sections were used for immunofluorescence and stored at − 80 °C.

### Immunofluorescence

Immunofluorescence was performed as described earlier with some modifications [31]. Briefly, brain sections were degreased in acetone for 20 min at 4 °C. After incubated with a blocking solution (10% normal goat serum, 0.3% Triton X-100 in PBS) at room temperature for 1 h, the sections were incubated with primary antibodies overnight at 4 °C. The following primary antibodies were used: mouse anti-6E10 monoclonal antibody (1:500; SIG-39300; Covance, Princeton, NJ), mouse anti-12F4 monoclonal antibody (1:500; SIG-39144; Covance), and rabbit anti-Iba-1 polyclonal antibody (1:200; 019-19741; Wako Chemicals, Japan). After rinsing in PBS, sections were incubated with secondary antibodies: Alexa Fluor-488-conjugated goat anti-rabbit IgG antibody (1:300; R11034; Invitrogen, Carlsbad, CA) and Alexa Fluor-594-conjugated goat anti-mouse IgG antibody (1:500; R11005; Invitrogen). The fluorescent imaging was visualized by using Olympus IX-81-inverted fluorescence microscope and FV1000 confocal laser scanning microscope (Olympus Corporation, Japan).

### Statistical analysis

**Fig. 1:**
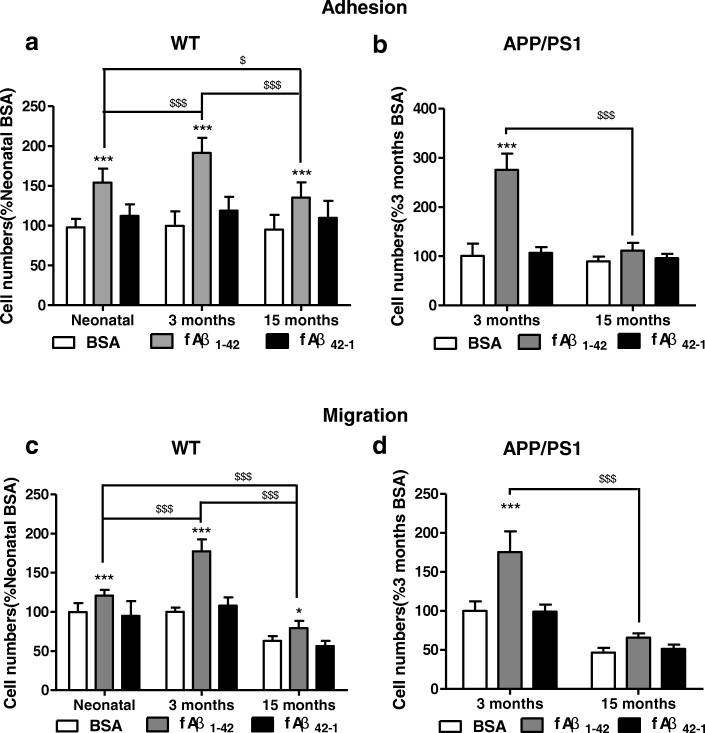
Aging led to decreased adhesion and migration of microglia to Aβ fibrils. Microglia from mice of various ages were isolated, and the effects of adhesion and migration of microglia to fAβ were determined using adhesion and migration assays. **a**, **b** The adhesion of microglia to fAβ. Microglia isolated from neonatal, 3-month-old, 15-month-old WT mice or 3-month-old, and 15-month-old APP/PS1 mice were plated on spots of BSA (0.01% in PBS), fAβ_1–42_ (10 μM) or fAβ_42–1_ (10 μM) for 24 h. The numbers of cells within spot areas were quantified. **a** WT mice. **b** APP/PS1 mice. **c**, **d** The migration of microglia to fAβ. Microglia isolated from neonatal, 3-month-old, 15-month-old WT mice or 3-month-old, and 15-month-old APP/PS1 mice were added to transwell membranes coated on their underside with BSA (0.01% in PBS), fAβ_1–42_ (10 μM), or fAβ_42–1_ (10 μM) for 24 h. The numbers of cells that transmigrated through the transwell membranes were quantified. **c** WT mice. **d** APP/PS1 mice. Values were reported as the mean ± SD, as a percentage of values determined in BSA group of neonatal mice (control, 100%). *p < 0.05, **p < 0.01, ***p < 0.001, when compared with the BSA group; ^*$*^*p* < 0.05, ^*$$$*^*p* < 0.001, *n* = 3

**Fig. 2:**
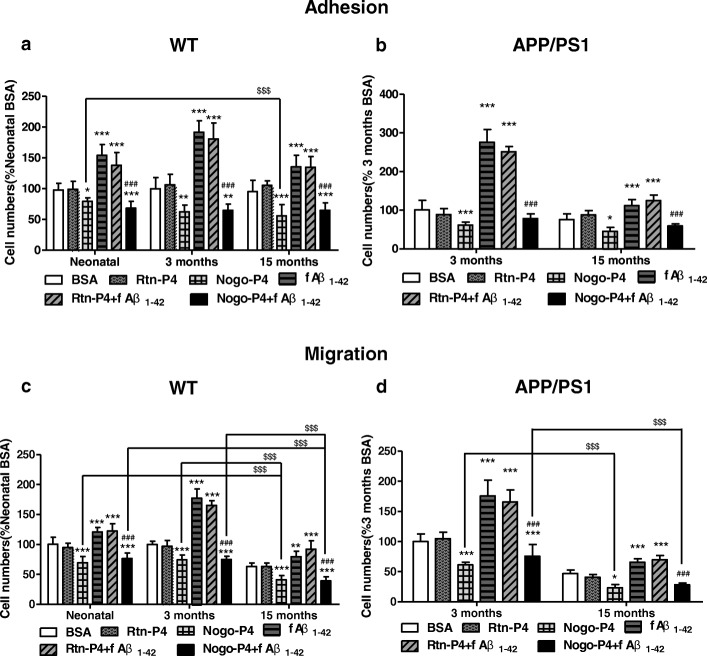
Adhesion and migration of microglia induced by Aβ fibrils were decreased by Nogo-P4. The effects of Nogo on the adhesion and migration of microglia isolated from WT and APP/PS1 mice in different ages to fAβ were measured. **a**, **b** Nogo-P4 inhibited adhesion of microglia to Aβ fibrils. Microglia isolated from neonatal, 3-month-old, 15-month-old WT mice or 3-month-old, and 15-month-old APP/PS1 mice were plated on spots of BSA (0.01% in PBS), Rtn-P4 (100 μg/ml), Nogo-P4 (100 μg/ml), fAβ_1–42_ (10 μM), Rtn-P4 (100 μg/ml) + fAβ_1–42_ (10 μM), or Nogo-P4 (100 μg/ml) + fAβ_1–42_ (10 μM) for 24 h. The numbers of cells within spot areas were quantified. **a** WT mice. **b** APP/PS1 mice. **c**, **d** Nogo-P4 inhibited the migration of microglia to Aβ fibrils. Microglia isolated from neonatal, 3-month-old, 15-month-old WT mice or 3-month-old, and 15-month-old APP/PS1 mice were added to transwell membranes coated on their underside with BSA (0.01% in PBS), Rtn-P4 (100 μg/ml), Nogo-P4 (100 μg/ml), fAβ_1–42_ (10 μM), Rtn-P4 (100 μg/ml) + fAβ_1–42_ (10 μM), or Nogo-P4 (100 μg/ml) + fAβ_1–42_ (10 μM) for 24 h. The numbers of cells that transmigrated through the transwell membranes were quantified. **c** WT mice. **d** APP/PS1 mice. Values were reported as the mean ± SD, as a percentage of values determined in BSA group of neonatal mice (control, 100%). **p < 0.01, ***p < 0.001, when compared with the BSA group; ^*###*^*p* < 0.001, when compared with fAβ_1–42_ group; ^*$*^*p* < 0.05, ^*$$*^*p* < 0.01, ^*$$$*^*p* < 0.001, *n* = 3

All present data represent the results of three independent experiments and repeats twice within each experiment. Statistical analysis was performed using one-way analysis of variance (ANOVA) with post hoc Tukey’s tests or Student’s *t* tests using GraphPad Prism 6 software (GraphPad Software Inc., La Jolla, CA, USA). Two-way ANOVAs followed by post hoc Tukey’s tests were utilized for multiple-factor comparisons of the parameters in Figs. 1 and 2. All data were presented as mean ± SD. *p* values of *p < 0.05 was considered statistically significant.

## Results

### Aging led to decreased adhesion and migration of microglia to Aβ fibrils

In AD, microglia migrates and adheres to Aβ plaque deposition [32–34], and the processes have been shown to participate in the clearance of Aβ plaques via phagocytosis and degradation of Aβ [35, 36]. The aggregation of fAβ_1–42_ was validated by the Congo Red dye binding assay (Additional file 1: Figure S1). To investigate effects of aging on adhesion and migration of microglia to Aβ fibrils, microglia from WT and APP/PS1 mice of various ages were isolated, and adhesion and migration of microglia to fAβ were determined. fAβ did not influence proliferation of microglia (Addtional file 2: Figure S2). Quantification of the data (Fig. 1a, b) showed that, compared with BSA spots, the numbers of microglia isolated from WT and APP/PS1 mice adhering to fAβ_1–42_ were increased. There was no significant change in the numbers of microglia isolated from WT and APP/PS1 mice adhering to fAβ_42–1_ (rev Aβ) spots (Fig. 1a, b). Aβ_42–1_ is the reverse peptide of Aβ_1–42_ and does not exert the activity of Aβ_1–42_. Corresponding with the results of the adhesion assay, the numbers of microglia isolated from WT and APP/PS1 mice that transmigrate to the underside of fAβ_1–42_ were increased when compared with the BSA-treated transwell (Fig. 1c, d). There was no significant change in the numbers of microglia isolated from WT and APP/PS1 mice migrating to fAβ_42–1_ (Fig. 1c, d). These data suggested that adhesion and migration of microglia were increased by fAβ_1–42_.

Compared with neonatal WT mice, adhesion and migration of microglia to fAβ_1–42_ were significantly increased in 3-month-old WT mice (Fig. 1a, c). Furthermore, adhesion and migration of microglia to fAβ_1–42_ were significantly decreased in 15-month-old WT mice (Fig. 1a, c). Consistent with the results in WT mice, adhesion and migration of microglia to fAβ_1–42_ were significantly decreased by aging in APP/PS1 mice (Fig. 2b, d). Taken together, these results implied that microglia derived from aged mice exhibited declines in the adhesion and migration to fAβ_1–42_.

As showed in Fig. 1c, d, the migration of microglia in BSA or fAβ_42–1_ significantly decreased in 15-month-old WT and APP/PS1 mice. It could be concluded that the migration, not the adhesion, of microglia in BSA and fAβ_42–1_ decreased with aging.

### The adhesion and migration of microglia induced by Aβ fibrils were decreased by the Nogo

It has been reported that Nogo-A is localized in the senile plaques of patients with AD [16], and the interaction of Nogo-66 and NgR inhibits adhesion and migration of microglia [22]. The effects of Nogo on the adhesion and migration of microglia isolated from WT and APP/PS1 mice in different ages to fAβ were measured. Nogo-66 is a 66-aa hydrophilic region of Nogo located between the two TM domains and has the inhibitory properties of Nogo-A. Nogo-P4 is a 25 aa inhibitory peptide sequence (residues 31–55 of Nogo-66), the active segment of Nogo-66, and has the core inhibitory activity of Nogo-66 [12]. These results in Fig. 2a, b showed that compared with BSA spots, the numbers of microglia isolated from WT and APP/PS1 mice adhering to Nogo-P4 were decreased and there was no significant change in the number of microglia adhering to Rtn-P4 spots, in which Rtn-P4 indicates as Reticulon 1 (Rtn1) non-inhibitory control/blocking peptide of Nogo-P4. Moreover, in contrast to the spots harboring BSA or fAβ_1–42_ alone, microglia isolated from WT and APP/PS1 mice did not adhere well to the spots containing Nogo-P4 and fAβ_1–42_ and Rtn-P4 had no effect (Fig. 2a, b), suggesting that Nogo-P4 has an anti-adherent effect on microglia to fAβ_1–42_. Next, migration of microglia isolated from WT and APP/PS1 mice was characterized and results showed that number of microglia that transmigrated to the underside of Nogo-P4 was reduced when compared with BSA-treated transwells (Fig. 2c, d). There was no significant change in the numbers of microglia migrating to Rtn-P4 (Fig. 2c, d). Moreover, Nogo-P4 significantly inhibited the migration of microglia to fAβ_1–42_ compared with BSA or fAβ_1–42_ groups and Rtn-P4 had no effect (Fig. 2c, d), indicating that Nogo-P4 could inhibit migration of microglia to fAβ. Furthermore, compared with neonatal WT mice, the inhibitory effects of Nogo on the adhesion of microglia were increased in 15-month-old WT mice (Fig. 2a), which was similar in APP/PS1 mice (Fig. 2b). Additionally, the inhibitory effect of Nogo on migration of microglia to fAβ_1–42_ was significantly increased with aging in WT mice and APP/PS1 mice (Fig. 2c, d). Taken together, these results suggested that Nogo decreased adhesion and migration of microglia to fAβ_1–42_ and the increased inhibitory effects could be observed in aging microglia isolated from WT and APP/PS1 mice.

**Fig. 3:**
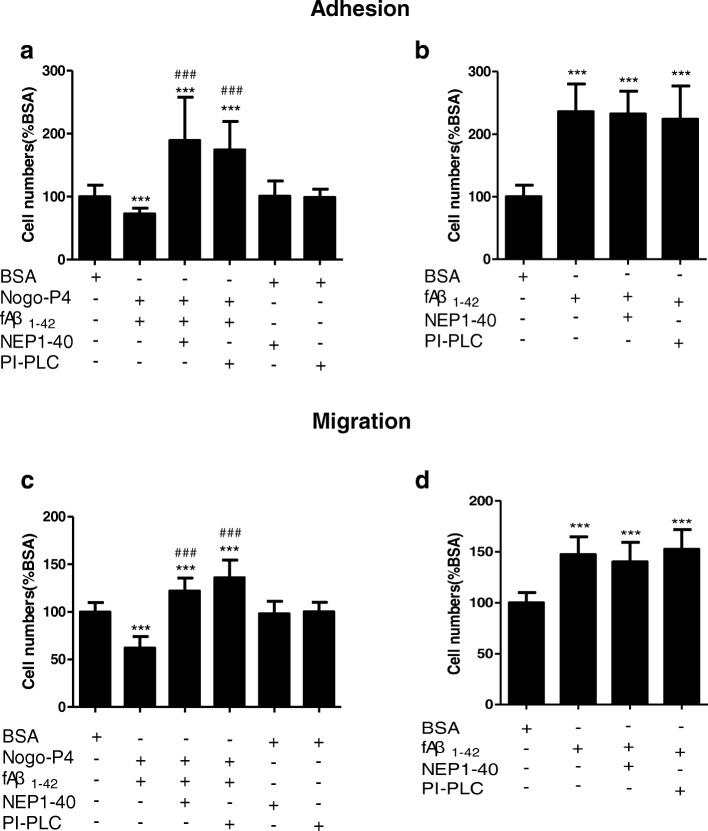
NgR mediated the effects of Nogo-P4 on adhesion and migration of adult microglia to fAβ. **a**, **b** NgR mediated the effect of Nogo-P4 on adhesion of adult microglia to fAβ_1–42_. Before adding to wells pre-coated with BSA (0.01% in PBS), fAβ_1–42_ (10 μM), or Nogo-P4 (100 μg/ml) + fAβ_1–42_ (10 μM), microglia from 3-month-old mice were pretreated with NEP1-40 (10 μM) or PI-PLC (0.3 U/ml) for 30 min to interrupt the function of NgR. The numbers of cells within spot areas were quantified. **c**, **d** NgR mediated the effect of Nogo-P4 on migration of adult microglia to fAβ_1–42_. Before adding into the transwells pre-coated with BSA (0.01% in PBS), fAβ_1–42_ (10 μM), or Nogo-P4 (100 μg/ml) + fAβ_1–42_ (10 μM), microglia from 3-month-old mice were pretreated with NEP1-40 (10 μM) or PI-PLC (0.3 U/ml) for 30 min to interrupt the function of NgR. The cell numbers transmigrated through transwell membranes were quantified. Values were reported as the mean ± SD, as a percentage of values determined in BSA group (control, 100%). *p < 0.05, **p < 0.01, ***p < 0.001, when compared with the BSA group; ^*###*^*p* < 0.001, when compared with the Nogo-P4 + fAβ_1–42_ group, *n* = 3

Rather than early postnatal microglia, adult microglia may provide a more relevant model for identifying the precise microglia-Aβ interaction during disease [37–40]. Adult microglia isolated from 3-month-old WT mice were chosen to study the further mechanism. Our previous research demonstrated that NgR is expressed on microglia [22] and that NgR protein expression is significantly increased in microglia with aging [25]. Next, we pretreated microglia with NEP1-40 (10 μM) or PI-PLC (0.3 U/ml) and then observed the effects of NgR on the inhibitory effects on the adhesion and migration of microglia to fAβ_1–42_. NEP1–40 is a competitive antagonist peptide derived from the first 40 amino acids of the Nogo-66 and promotes axonal outgrowth through blocking the binding of Nogo-66 to NgR [41]. The enzymatic action of PI-PLC results in the release of GPI anchored NgR from the cell surface [14]. As shown (Fig. 3a, c), the effects of Nogo-P4 on adult microglial adherence and migration to fAβ_1–42_ was significantly reversed by NEP1-40 or PI-PLC treatment. However, NEP1-40 or PI-PLC treatment alone had no effects in microglial adhesion and migration induced by fAβ_1–42_ (Fig. 3b, d). These results suggest that the Nogo/NgR pathway took part in the effects of fAβ_1–42_ on the microglial adhesion and migration.

### Rho GTPases were involved in the inhibitory effects of Nogo on microglial adhesion and migration to Aβ fibrils

**Fig. 4:**
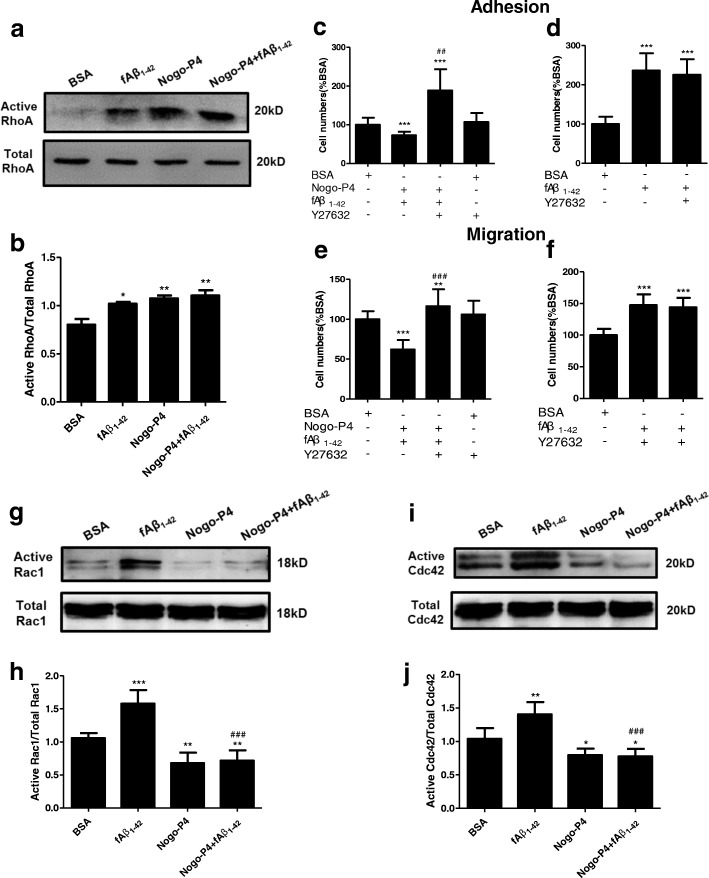
Rho GTPases were involved in effects of Nogo-P4 on microglia adhesion and migration to fAβ. **a**, **b** The activation of RhoA in microglia was determined using a Rho Activation Assay Kit after microglia from 3-month-old mice were stimulated with BSA (0.01% in PBS), fAβ_1–42_ (10 μM), Nogo-P4 (100 μg/ml), or Nogo-P4 (100 μg/ml) + fAβ_1–42_ (10 μM) for 6 h. Values were reported as the mean ± SD. *p < 0.05, **p < 0.01, when compared with BSA group, *n* = 3. **c**–**f** RhoA/ROCK pathway involved in the regulation of Nogo-P4 on adhesion and migration of microglia from 3-month-old mice to Aβ fibrils. Quantitative assessment of cell adhesion (**c**, **d**) and migration (**e**, **f**) by determining the cell numbers, after pre-treatment with Y27632 (50 μM) for 30 min. Values were reported as the mean ± SD, as a percentage of values determined in BSA group (control, 100%). **p < 0.01, ***p < 0.001, when compared with the BSA group; ^*##*^*p* < 0.01, when compared with the Nogo-P4 + fAβ_1–42_ group, *n* = 3. The activation of Rac1 (**g**, **h**) and Cdc42 (**i**, **j**) in microglia were determined using a Rac1/Cdc42 Activation Assay Kit. Microglia from 3-month-old mice were stimulated with BSA (0.01% in PBS), fAβ_1–42_ (10 μM), Nogo-P4 (100 μg/ml), or Nogo-P4 (100 μg/ml) + fAβ_1–42_ (10 μM) for 6 h. Values were reported as the mean ± SD. **p < 0.01, ***p < 0.001, when compared with the BSA group; ^*###*^*p* < 0.001, when compared with the fAβ_1–42_ group, *n* = 3

Rho GTPases including Rho, Rac1, and Cdc42 are best known as regulators of the actin cytoskeleton, cell polarity, microtubule dynamics, and vesicular trafficking [42], which participate in the processes of cell adhesion and migration. Binding of Nogo-66 with NgR could activate RhoA and its downstream molecule ROCK, which contributes to adhesion and migration of microglia [22]. To determine whether RhoA acts as an intracellular signal transducer for effects of Nogo-P4 on microglial adherence and migration to fAβ_1–42_, RhoA activity was measured. The amount of activated RhoA of adult microglia in fAβ_1–42_, Nogo-P4, or Nogo-P4 + fAβ_1–42_-coated wells was higher than BSA substrate-coated well (Fig. 4a, b). To investigate the role of the RhoA/ROCK pathway on the effects of Nogo-P4 on adhesion and migration of microglia to fAβ_1–42_, adult microglia were pre-treated with Y27632 (50 μM) to block the function of ROCK before performing the adhesion or migration assay. Y27632 is widely used as a specific inhibitor of ROCK [43, 44]. The results (Fig. 4c–f) showed that treatment with Y27632 alone did not significantly affect the increased adhesion and migration of adult microglia induced by fAβ_1–42_. Y27632 pre-treatment exhibited obvious conversion effects of Nogo-P4 on adult microglial adherence and migration to fAβ_1–42_ (Fig. 4c–f). The data above implying that the RhoA/ROCK pathway is involved in the inhibited effects of Nogo-P4 on microglial adhesion and migration to fAβ_1–42_.

Cdc42 and Rac1 regulate the direction of migration, and RhoA promotes actin: myosin contractions in the cell body and at the rear [42]. The effects of fAβ_1–42_ and Nogo-P4 on the activity of Cdc42 and Rac1 in adult microglia were measured. The results in Fig. 4g–j showed that fAβ_1–42_ promoted the activation of Cdc42 and Rac1, while Nogo-P4 decreased the activation of Cdc42 and Rac1 in adult microglia. Moreover, Nogo-P4 inhibited the activity of Cdc42 and Rac1 induced by fAβ_1–42_ in adult microglia (Fig. 4g–j). These results indicated that activation of Cdc42 and Rac1 induced by fAβ_1–42_ were decreased by Nogo-P4, which may contribute to the effects of Nogo-P4 on adhesion and migration of microglia to fAβ.

### Nogo/NgR pathway regulated microglia spreading and membrane protrusion formation induced by Aβ fibrils

**Fig. 5:**
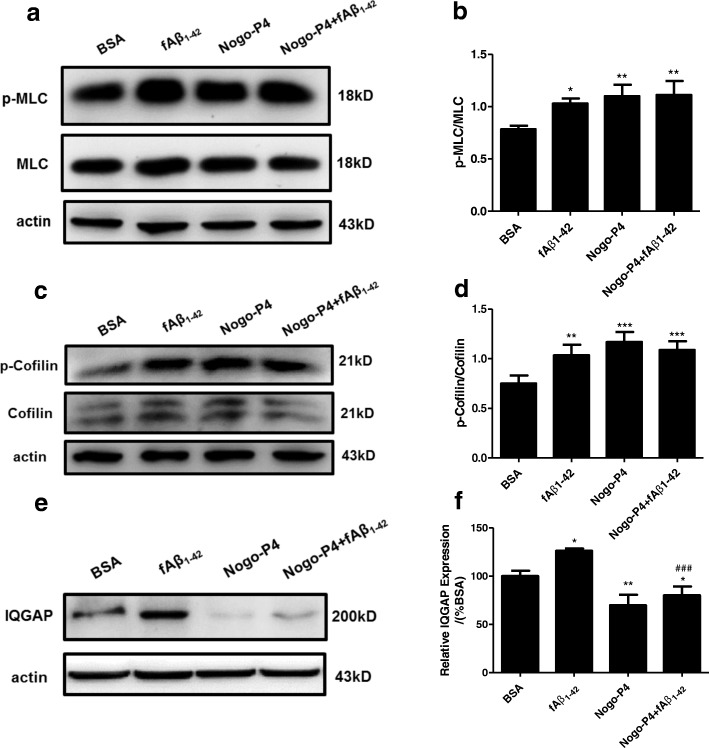
Effects of Nogo-P4 on phosphorylation of MLC and cofilin and expression of IQGAP in microglia. MLC, cofilin, and IQGAP are key cytoskeleton proteins. After microglia from 3-month-old mice were treated with BSA (0.01% in PBS), fAβ_1–42_ (10 μM), Nogo-P4 (100 μg/ml), or Nogo-P4 (100 μg/ml) + fAβ_1–42_ (10 μM) for 6 h, phosphorylation of MLC and cofilin, and expression of IQGAP were quantified using Western blot. MLC (**a**, **b**), cofilin (**c**, **d**). Values were reported as the mean ± SD. IQGAP (**e**, **f**). Values were reported as the mean ± SD, as a percentage of values determined in the BSA group (control, 100%). *p < 0.05, **p < 0.01, ***p < 0.001, when compared with the BSA group; ^*###*^*p* < 0.001, when compared with the fAβ_1–42_ group, *n* = 3

Cytoskeleton reorganization is closely related to cell adhesion and migration. Rho GTPases are the critical mediators in regulating the formation of distinct actin filament-containing structures, which is mediated by downstream molecules such as myosin-regulatory light chain (MLC) and cofilin [45]. Western blot results showed that fAβ_1–42_, Nogo-P4 or Nogo-P4, and fAβ_1–42_ significantly promoted phosphorylation levels of MLC (Fig. 5a, b) and cofilin (Fig. 5c, d). The scaffolding protein IQGAP, which can be activated by either Cdc42 or Rac, might be involved in cell migration [46]. Compared with BSA, expression of IQGAP was increased by fAβ_1–42_ and was decreased by Nogo-P4 in adult microglia (Fig. 5e, f). Moreover, Nogo-P4 inhibited expression of IQGAP induced by fAβ_1–42_ in adult microglia (Fig. 5e, f).

**Fig. 6:**
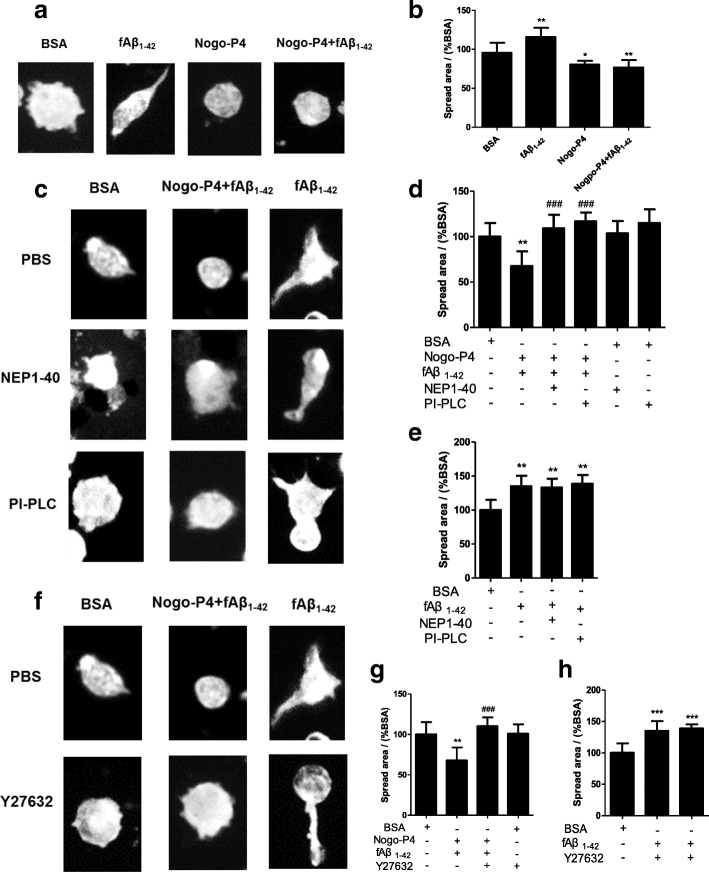
RhoA/ROCK pathway contributed to regulation of Nogo/NgR pathway on microglia spread and membrane protrusion formation. To explore the effect of Nogo-P4 on cytoskeleton reorganization of microglia and the associated mechanism, F-actin staining and the mean spread area of the microglia were examined. **a**, **b** Nogo-P4 inhibited microglia spread and membrane protrusion formation induced by Aβ fibrils. Microglia from 3-month-old mice were plated in 96-well plates pre-coated with BSA (0.01% in PBS), fAβ_1–42_ (10 μM), Nogo-P4 (100 μg/ml), or Nogo-P4 (100 μg/ml) + fAβ_1–42_ (10 μM) for 8 h. **a** Photomicrographs of microglia stained with rhodamine-conjugated phalloidin. **c**–**e** The effects of Nogo-P4 on spread and membrane protrusion formation induced by Aβ fibrils were mediated by NgR. Before adding to the wells pre-coated with BSA (0.01% in PBS), fAβ_1–42_ (10 μM) or Nogo-P4 (100 μg/ml) + fAβ_1–42_ (10 μM), microglia from 3-month-old mice were pre-treated with NEP1-40 (10 μM) or PI-PLC (0.3 U/ml) for 30 min to interrupt the function of NgR. **c** Photomicrographs of microglia stained with rhodamine-conjugated phalloidin. **d**, **e** Quantitative assessment of cell spreading by determining the spread area of the microglia. **f**–**h** RhoA/ROCK pathway contributed to the regulation of Nogo-P4 on spread and membrane protrusion formation induced by Aβ fibrils. After pre-treatment with PBS or Y27632 (50 μM) for 30 min, microglia from 3-month-old mice were plated in 96-well plates pre-coated with BSA (0.01% in PBS), fAβ_1–42_ (10 μM), or Nogo-P4 (100 μg/ml) + fAβ_1–42_ (10 μM) for 8 h. **f** Photomicrographs of microglia stained with rhodamine-conjugated phalloidin. Values were reported as the mean ± SD, as a percentage of values determined in BSA group (control, 100%). *p < 0.05; **p < 0.01; ***p < 0.01, when compared with the BSA group, *n* = 3. ^*###*^*p* < 0.001, when compared with the Nogo-P4 + fAβ_1–42_ group, *n* = 3

To further explore the effect of Nogo-P4 on cytoskeleton reorganization of microglia and associated mechanism, F-actin staining and the mean spread area in the microglia were examined. Adult microglia stimulated by BSA showed multiple membrane protrusions and spread normally, and fAβ_1–42_ promoted more membrane protrusions and the spread of cells, while Nogo-P4 or Nogo-P4 + fAβ_1–42_ induced a rapid rounding up of adult microglia that had lost polarization and lacked processes and protrusions (Fig. 6a). The mean spread area from adult microglia was significantly reduced in Nogo-P4 compared with BSA treatment (Fig. 6b). Moreover, compared with fAβ_1–42_ treatment, mean spread area induced by fAβ_1–42_ was decreased by Nogo-P4 (Fig. 6b), which was consistent with the insufficient adhesion and migration of microglia to fAβ_1–42_ after expose to Nogo-P4. Furthermore, NEP1-40 or PI-PLC pre-treatment could convert the effects of Nogo-P4 on membrane protrusion and the spread areas of adult microglia induced by fAβ_1–42_ (Fig. 6c, d). Y27632 pre-treatment could attenuate effects of Nogo-P4 on membrane protrusion and spread areas of adult microglia induced by fAβ_1–42_ (Fig. 6f, g). Moreover, NEP1-40 or PI-PLC (Fig. 6c, e) or Y27632 (Fig. 6f, h) treatment alone had no effect on cytoskeleton reorganization of microglia induced by fAβ_1–42_. This phenomenon implied that Nogo binding with NgR in adult microglia varied activation of Rho GTPase and downstream molecules such as MLC, cofilin, and IQGAP; further restricted the protrusions extension and cell polarization; and finally resulted in decreased microglial migration and adhesion to Aβ fibrils.

With aging, the increased expression of NgR in microglia has been reported in our previous study [25]. The expression of RhoA in microglia during aging was measured by Western blot, and F-actin staining and mean spread area in microglia during aging were examined. The results showed that with aging, expression of RhoA were significantly increased in 3-month-old and 15-month-old microglia (Additional file 3: Figure S3 A, B). There is no difference in expression of 3-month-old and 15-month-old microglia (Additional file 3: Figure S3 A, B). In addition, compared with neonatal and 3-month-old microglia, mean spread area was reduced in 15-month-old microglia (Additional file 3: Figure S3 C, D). Taken together, these results implied that microglia derived from aged mice exhibited increase in the expression of NgR and RhoA and decline in the protrusion extension and cell polarization.

### Inhibition of the Nogo/NgR pathway enhanced microglial recruitment toward Aβ deposits and CD36 expression in APP/PS1 mice

**Fig. 7:**
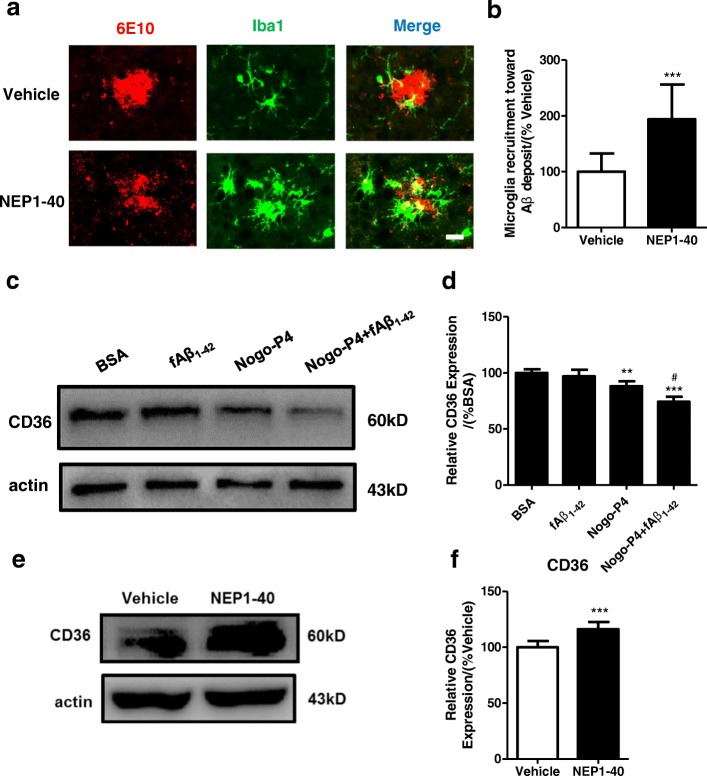
Blocking of Nogo/NgR pathway enhanced microglial recruitment toward Aβ and CD36 expression in APP/PS1 mice. To determine the effects of the Nogo/NgR pathway on adhesion and migration of microglia to Aβ fibrils in vivo, NEP1-40 was intracerebroventricularly administered to APP/PS1 mice for 2 months using continuous an Alzet mini-pump. **a**, **b** Mice brain sections were processed for anti-Aβ and anti-Iba1 immunofluorescence as indicated. Bar = 50 μM. **b** Ten randomly chosen plaque areas in the cortex and hippocampus were evaluated for Iba1/Aβ colocalization per animal. Values were reported as the mean ± SD, as a percentage of values determined in vehicle group (control, 100%). ***p < 0.01, when compared with the vehicle group, *n* = 4–5. **c**, **d** After microglia from 3-month-old mice were treated with BSA (0.01% in PBS), fAβ_1–42_ (10 μM), Nogo-P4 (100 μg/ml), or Nogo-P4 (100 μg/ml) + fAβ_1–42_ (10 μM) for 24 h, expression of CD36 was measured by Western blot. Values were reported as the mean ± SD, as a percentage of values determined in BSA group (control, 100%). **p < 0.01; ***p < 0.01, when compared with the BSA group; ^*#*^*p* < 0.05, when compared with the fAβ_1–42_ group, *n* = 3. **e**, **f** NEP1-40 was intracerebroventricularly administered to APP/PS1 mice for 2 months. Homogenates from brain tissue of APP/PS1 transgenic mice were subjected to Western blot analysis with markers of CD36. Values were reported as the mean ± SD, as a percentage of values determined in vehicle group (control, 100%). ***p < 0.01, when compared with the vehicle group, *n* = 4–5

To determine the effects of the Nogo/NgR pathway on adhesion and migration of microglia to fAβ in vivo, NEP1-40 was intracerebroventricularly administered by continuous infusion into APP/PS1 mice aged from 6 to 8 months old with an Alzet mini-pump. Therefore, recruitment of microglia toward Aβ deposits was determined in APP/PS1 mice. To analyze plaque-associated microglia, confocal analysis of brain sections stained with 6E10 for Aβ plaques and Iba-1 for microglia was determined. As observed (Fig. 7a, b), more Iba-1^+^ microglia were recruited to the Aβ plaques in the NEP1-40 group than in the Vehicle group, which was consistent with our previous finding in vitro. To confirm these results, the 12F4 antibody [47, 48] (raised against Aβ_36–43_, specific for Aβ42) was used for Aβ in immunofluorescence. As showed (Additional file 4: Figure S4), more Iba-1^+^ microglia were recruited to the 12F4^+^ Aβ in the NEP1-40 group than in the vehicle group, which was consistent with our previous finding in 6E10 immunofluorescence.

CD36 has been suggested as an important receptor for microglial Aβ phagocytosis [49, 50] and also plays a pivotal role in the adhesion [51] and migration of microglia to fAβ [52]. To measure whether Nogo-P4 affects expression of CD36 in microglia, adult microglia were added into the wells pre-coated with BSA, fAβ_1–42_, Nogo-P4, or Nogo-P4+fAβ_1–42_, and cell lysates were collected for Western blot assays. The data showed that Nogo-P4 or Nogo-P4 + fAβ_1–42_ significantly attenuated the expression of CD36 in microglia (Fig. 7c, d), indicating that CD36 may involve in the inhibitory effects of Nogo-P4 on adhesion and migration to Aβ fibrils. Corresponding to the experiments in vitro, NEP1-40 increased expression of CD36 in APP/PS1 mice brain (Fig. 7e, f). The results above suggest that the inhibition of the Nogo/NgR pathway promotes microglial recruitment to the Aβ plaques and expression of key phagocytosis receptor and might further increased clearance of amyloid load in APP/PS1 mice.

## Discussion

**Fig. 8:**
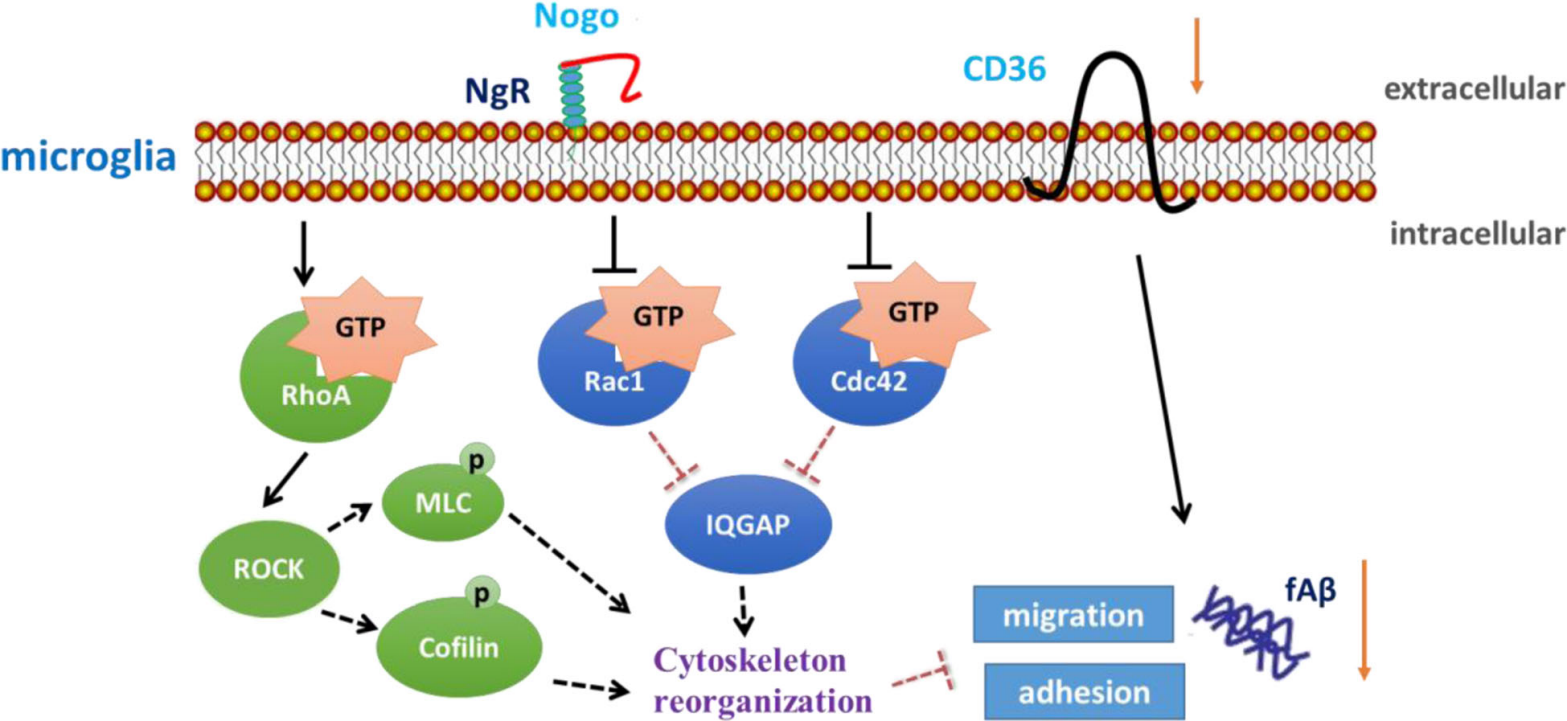
Schematic diagram of the effects of Nogo/NgR pathway on microglial adhesion and migration to fAβ. The interaction of Nogo with NgR enhanced activity of RhoA and phosphorylation of MLC and cofilin and decreased activity of Rac1 and Cdc42 and expression of IQGAP, which regulated cytoskeleton arrangement. Then, adhesion and migration of microglia to fAβ was decreased. Moreover, expression of CD36 in microglia was decreased by the Nogo/NgR pathway, which might also contribute to the downregulation of microglial adhesion and migration to fAβ

In this study, we have found that microglia exhibited decreased adhesion and migration to fAβ_1–42_ by aging. Moreover, the Nogo/NgR pathway inhibited the adhesion and migration of microglia to fAβ_1–42_ through cytoskeleton reorganization mediated by Rho GTPases (Fig. 8).

From results presented above, three points are particularly noteworthy. Firstly, with aging, the adhesion and migration of microglia to Aβ fibrils were decreased and the Nogo might inhibit adhesion and migration of microglia to Aβ fibrils. Recent studies have indicated that along with aging and the process of disease, the ability of microglia to specifically phagocytose Aβ fibrils is reduced in aged mice [10, 11]. Because microglial phagocytosis of Aβ is a complex process, in this study, the ability of microglia to adhere and migrate to Aβ was dissected. We found that microglia derived from aging mice exhibited decreased adhesion and migration to fAβ_1–42_, which could at least explain reduction in the phagocytosis of Aβ fibrils by microglia with aging. It is known that Nogo-A is localized with Aβ plaques in patients with AD and APP/PS1 mice, and our previous study indicated that the expression of NgR is enhanced in microglia with aging [25]. Moreover, binding of NgR with Nogo-66 inhibits microglial adhesion and migration [22]. Our data showed that Nogo-P4 (the 25-amino acid inhibitory peptide sequence of Nogo-66) could inhibit microglial ability to adhere and migrate fAβ. Moreover, we found that increased in the inhibitory effects of Nogo-P4 on the migration of microglia was exhibited in aged WT and APP/PS1 mice. Furthermore, blocking binding of Nogo-P4 to NgR on microglia by NEP1–40 or PI-PLC pre-treatment rescued the inhibition of Nogo-P4 on the adhesion and migration of microglia to fAβ. These data indicate that Nogo/NgR pathway contributed to the effects of fAβ_1–42_ on the microglial adhesion and migration. Hence, the Nogo/NgR pathway might contribute to the age-associated decrease in the ability of microglia to interact with Aβ fibrils.

Secondly, cytoskeleton arrangement mediated by Rho GTPases was responsible for the effects of Nogo on adhesion and migration of microglia to Aβ fibrils. It has been reported that Rho GTPase family, which is downstream of the Nogo/NgR pathway [22, 53], can regulate cytoskeleton arrangement during cell migration [54, 55]. For example, Rho regulates the assembly of contractile, actin, and myosin filaments, while Rac and Cdc42 regulate polymerization of actin to form peripheral lamellipodial and filopodial protrusions, respectively. Notably, Cdc42 is required for the establishment of cell polarity by affecting the microtubule cytoskeleton and gene transcription [56]. Moreover, Rac1 is required at the front of the cell to regulate actin polymerization and membrane protrusion [57]. Our previous study confirmed that interaction of Nogo-66 and NgR triggered the activation of RhoA, and the RhoA/ROCK pathway mediated the effects of Nogo-66 on cell adhesion, migration, and spreading [22] (Additional file 2). In this study, we found that fAβ_1–42_, Nogo-P4, or Nogo-P4 + fAβ_1–42_ could active RhoA in adult microglia and that the RhoA/ROCK pathway was involved in the inhibitory effects of Nogo-P4 on adhesion and migration of microglia to fAβ. Furthermore, a recently study found that enhancement in actin polymerization induced by fAβ was associated with increased activity of Rac1 and Cdc42 in hippocampal neurons [58]. Nogo-66 also decreased Cdc42 activity in microglia [22]. Our observation also indicated that activity of Rac1 and Cdc42 was induced by fAβ; meanwhile, the activity was inhibited by Nogo-P4, which further mediated the regulation of the protrusions extension and cell polarization in adult microglia.

Key components of stress fibers include F-actin and myosin; meanwhile, phosphorylated MLC (p-MLC) induces motor activity in myosin. The Rho/ROCK pathway may induce motor activity in myosin by phosphorylating MLC [59], and the Rho/ROCK/MLC/myosin pathway is a crucial determinant for amoeboid motility [60]. Moreover, Nogo-66 signals regulate phosphorylation profile of actin depolymerization factor cofilin, which is involved by RhoA/ROCK pathway [61]. Cofilin could regulate myelin phagocytosis of microglia through its ability to control remodeling of F-actin [62]. Furthermore, IQGAP scaffolding effectors of Cdc42 and Rac [63] may regulate cell polarity and membrane protrusion through actin polymerization, which participates in cell migration [64, 65]. Our results indicated that Nogo-P4 promotes phosphorylation of MLC and cofilin and inhibited expression of IQGAP induced by fAβ in adult microglia. Moreover, the Nogo/NgR pathway restricted protrusions extension and cell polarization induced by fAβ in adult microglia, which was mediated by Rho GTPases.

Thirdly, our observations were proven in APP/PS1 transgenic mice, a common AD animal model. Consistent with our study in vitro, blocking the Nogo/NgR pathway by NEP1-40 significantly induced recruitment of microglia toward Aβ deposits. NEP1-40, a competitive antagonist peptide of Nogo/NgR pathway, has been previously shown to promote axon regeneration and improve outcomes after spinal cord injury and stroke in vivo [66, 67]. Moreover, our previous study confirmed that the blockage of the Nogo/NgR signal pathway by NEP1-40 in microglia alleviates the formation of Aβ plaques in APP/PS1 transgenic mice [24]. The observation that recruitment of microglia toward Aβ deposits was increased by NEP1-40 suggests that microglia may phagocytose Aβ easier. CD36 has been suggested as an important receptor for microglial Aβ phagocytosis [50] and also contributes to the adhesion and migration of microglia to Aβ [51, 52, 68]. Some researchers have demonstrated that RhoA participates in expression of CD36 in macrophages [69] and monocytes [70]. In this study, we found that Nogo-P4 significantly attenuated the expression of CD36 in adult microglia, indicating that CD36 may contribute to inhibition of the Nogo/NgR pathway on adhesion and migration to fAβ. Moreover, blocking of the Nogo/NgR pathway promoted expression of CD36 which might increase clearance of Aβ and further ameliorate amyloid load in APP/PS1 mice (Additional file 4).

## Conclusion

In summary, the Nogo/NgR pathway could take part in Aβ pathology in AD by modulating microglial adhesion and migration to Aβ. Microglia derived from aging mice exhibits decreased adhesion and migration to fAβ_1–42_. The interaction of Nogo with NgR inhibits adhesion and migration of microglia to fAβ_1–42_, and Rho GTPases contribute to these effects by regulating cytoskeleton arrangement. Ablation of the Nogo/NgR pathway by NEP1-40 facilitates recruitment of microglia toward Aβ deposits and expression of CD36 in AD mice. These results provide a better understanding of relationship between the Nogo/NgR pathway and interaction of microglia with Aβ and may potentially aid in the development of treatments for AD progression.

## Additional files

Additional file 1: **Figure S1.** Congo Red dye binding assay of fAβ1–42. The aggregation of fAβ1–42 was validated by the Congo Red dye binding assay. (A) The relative Cb of fAβ1–42. (B) Photomicrographs of fAβ1–42. *p < 0.05; **p < 0.01, when compared with 0 μM fAβ1–42, *n* = 3. (PDF 120 kb)

Additional file 2: **Figure S2.** The locally proliferated microglia. (A) The locally proliferated microglia were quantified using IF staining of Ki67. (B) The ratio of Ki67+ cells/total cells (in %). Values were reported as mean ± SD. (PDF 458 kb)

Additional file 3: **Figure S3.** The expression of RhoA and the cytoskeleton in microglia during aging. (A-B) The expression of RhoA in microglia during aging were quantified using Western blot. Values were reported as the mean ± SD, as a percentage of values determined in the neonatal group (control, 100%). (C, D) To explore the effect of aging on cytoskeleton reorganization of microglia, F-actin staining and mean spread area of the microglia were examined. F Photomicrographs of microglia stained with rhodamine-conjugated phalloidin. Values were reported as mean ± SD, as a percentage of values determined in neonatal group (control, 100%). *p < 0.05, **p < 0.01, ***p < 0.001, when compared with neonatal group. (PDF 64 kb)

Additional file 4: **Figure S4.** The recruit of 12F4+ Aβ to microglia in APP/PS1 mice. Mice brain sections were processed for anti-12F4 and anti-Iba1 immunofluorescence as indicated. Bar = 50 μM. B: Ten randomly chosen plaque areas in the cortex and hippocampus were evaluated for Iba1/12F4+ Aβ colocalization per animal. Values were reported as the mean ± SD, as a percentage of values determined in vehicle group (control, 100%). ***p < 0.01, when compared with the vehicle group, *n* = 3–6. (PDF 71 kb)

## Abbreviations

AD: Alzheimer’s disease
Aβ: β-amyloid
fAβ: Fibrillar Aβ
MLC: Myosin-regulatory light chain
NFTs: Neurofibrillary tangles
NgR: Nogo receptor
PFA: Paraformaldehyde
