# Supplementary figures and images for: The adhesion and migration of microglia to β-amyloid (Aβ) is decreased with aging and inhibited by Nogo/NgR pathway
Source: J Neuroinflammation. 2018 Jul 20;15:210. doi: 10.1186/s12974-018-1250-1 (PMC6054753; doi:10.1186/s12974-018-1250-1)

# Additional file 1: Figure S1

A

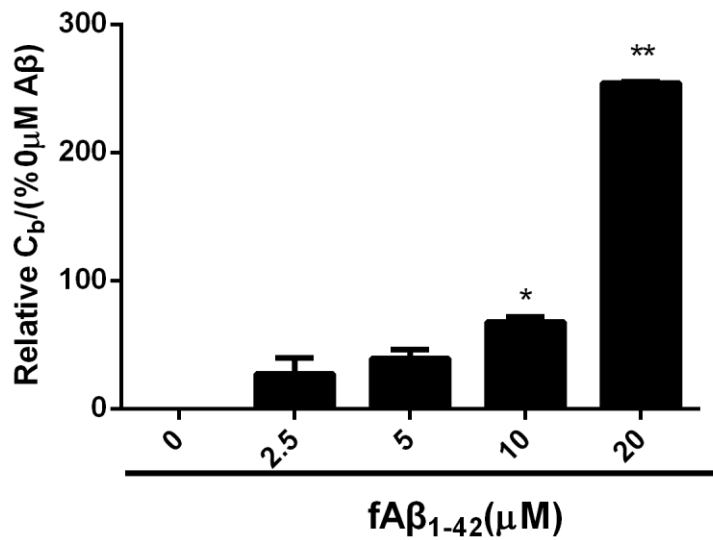

B

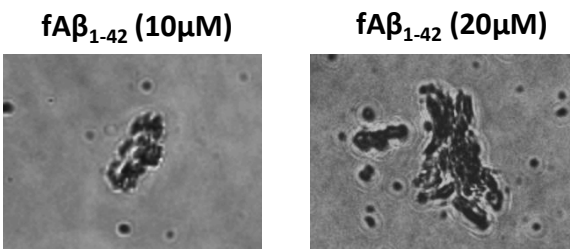

Supplement: Supplementary file 1 — Figure S1. Congo Red dye binding assay of fAβ1–42. The aggregation of fAβ1–42 was validated by the Congo Red dye binding assay. (A) The relative Cb of fAβ1–42. (B) Photomicrographs of fAβ1–42. *p < 0.05; **p < 0.01, when compared with 0 μM fAβ1–42, n = 3. (PDF 120 kb) [file 12974_2018_1250_MOESM1_ESM.pdf]

## Additional file 2: Figure S2

A

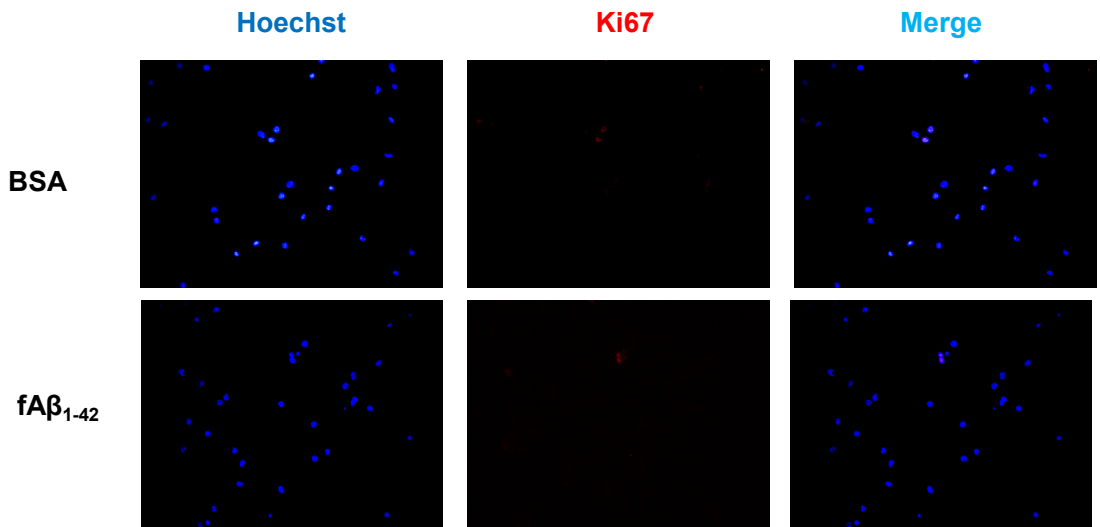

B

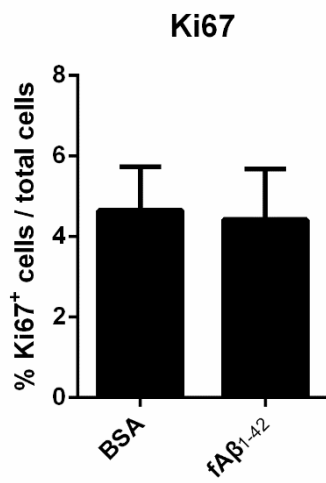

Supplement: Supplementary file 2 — Figure S2. The locally proliferated microglia. (A) The locally proliferated microglia were quantified using IF staining of Ki67. (B) The ratio of Ki67+ cells/total cells (in %). Values were reported as mean ± SD. (PDF 458 kb) [file 12974_2018_1250_MOESM2_ESM.pdf]

# Additional file 3: Figure S3

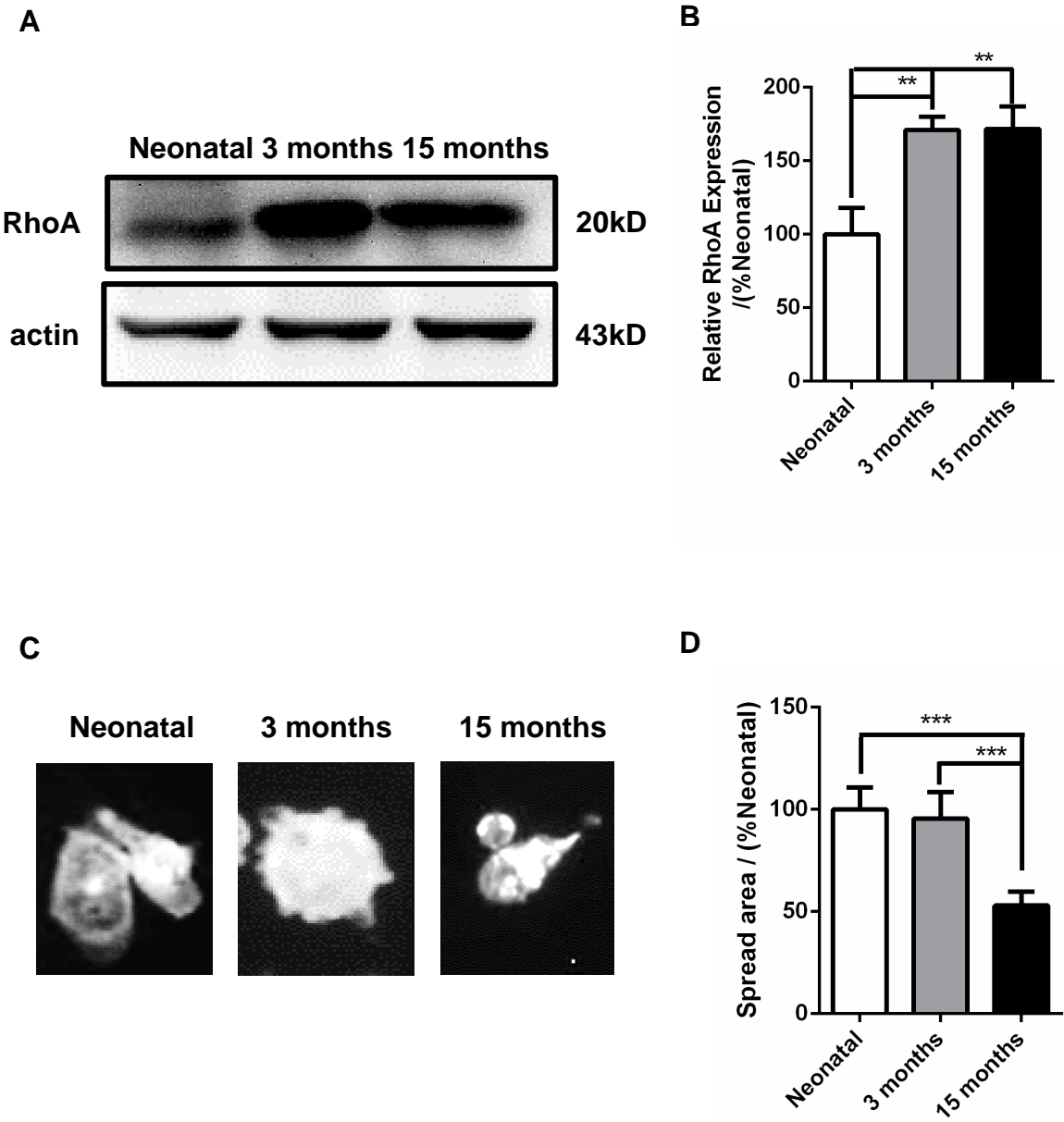

Supplement: Supplementary file 3 — Figure S3. The expression of RhoA and the cytoskeleton in microglia during aging. (A-B) The expression of RhoA in microglia during aging were quantified using Western blot. Values were reported as the mean ± SD, as a percentage of values determined in the neonatal group (control, 100%). (C, D) To explore the effect of aging on cytoskeleton reorganization of microglia, F-actin staining and mean spread area of the microglia were examined. F Photomicrographs of microglia stained with rhodamine-conjugated phalloidin. Values were reported as mean ± SD, as a percentage of values determined in neonatal group (control, 100%). *p < 0.05, **p < 0.01, ***p < 0.001, when compared with neonatal group. (PDF 64 kb) [file 12974_2018_1250_MOESM3_ESM.pdf]

# Additional file 4: Figure S4

A

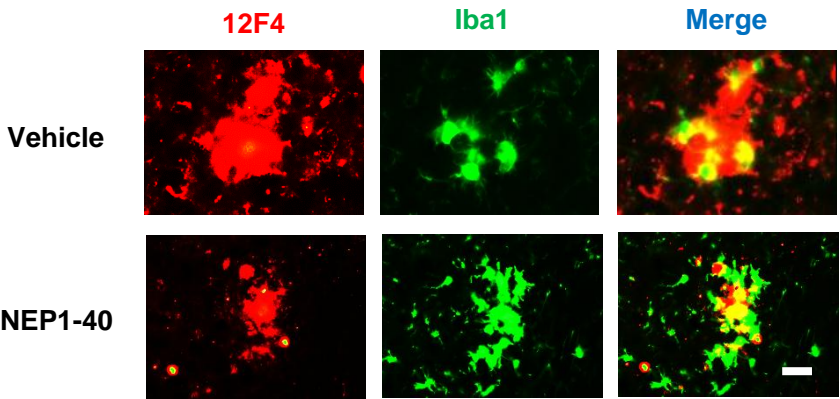

B

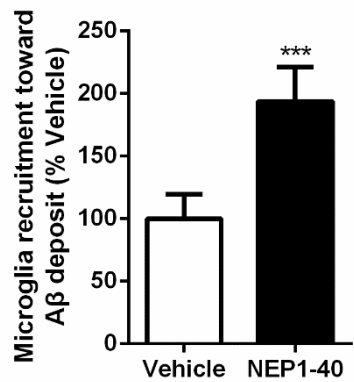

Supplement: Supplementary file 4 — Figure S4. The recruit of 12F4+ Aβ to microglia in APP/PS1 mice. Mice brain sections were processed for anti-12F4 and anti-Iba1 immunofluorescence as indicated. Bar = 50 μM. B: Ten randomly chosen plaque areas in the cortex and hippocampus were evaluated for Iba1/12F4+ Aβ colocalization per animal. Values were reported as the mean ± SD, as a percentage of values determined in vehicle group (control, 100%). ***p < 0.01, when compared with the vehicle group, n = 3–6. (PDF 71 kb) [file 12974_2018_1250_MOESM4_ESM.pdf]
